# Bis(guanidinium) 4,5-dichloro­phthalate monohydrate

**DOI:** 10.1107/S1600536811021192

**Published:** 2011-06-11

**Authors:** Graham Smith, Urs D. Wermuth

**Affiliations:** a Faculty of Science and Technology, Queensland University of Technology, GPO Box 2434, Brisbane, Queensland 4001, Australia

## Abstract

In the structure of the title hydrated salt, 2CH_6_N_3_^+^·C_8_H_2_Cl_2_O_4_^2−^·H_2_O, the planes of the carboxyl­ate groups of the dianion are rotated out of the plane of the benzene ring [dihedral angles = 48.42 (10) and 55.64 (9)°]. A duplex-sheet structure is formed through guanidinium–carboxyl­ate N—H⋯O, guanidinium–water N—H⋯O and water–carboxyl­ate O—H⋯O hydrogen-bonding associations.

## Related literature

For the structures of 1:1 salts of 4,5-dichloro­phthalate, see: Mallinson *et al.* (2003 ▶); Bozkurt *et al.* (2006 ▶); Smith *et al.* (2008 ▶, 2009 ▶); Smith & Wermuth (2010*a* ▶,*d* ▶). For 1:2 salts, see: Büyükgüngör & Odabaşoğlu (2007 ▶); Smith & Wermuth (2010*a* ▶,*c* ▶). For guanidinium salts of aromatic dicarb­oxy­lic acids, see: Krumbe & Haussuhl (1986 ▶); Smith & Wermuth (2010*b* ▶).
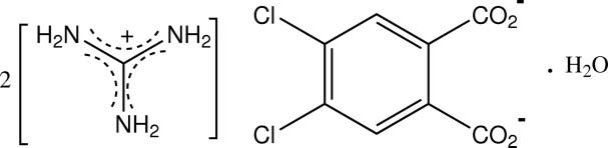


## Experimental

### 

#### Crystal data

2CH_6_N_3_^+^·C_8_H_2_Cl_2_O_4_^2−^·H_2_O
*M* *_r_* = 371.19
Monoclinic, 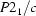

*a* = 15.9797 (5) Å
*b* = 6.9432 (2) Å
*c* = 15.2266 (5) Å
β = 94.650 (3)°
*V* = 1683.84 (9) Å^3^
*Z* = 4
Mo *K*α radiation
μ = 0.42 mm^−1^
*T* = 200 K
0.28 × 0.25 × 0.20 mm

#### Data collection

Oxford Diffraction Gemini-S CCD area-detector diffractometer
Absorption correction: multi-scan (*CrysAlis PRO*; Oxford Diffraction, 2010 ▶) *T*_min_ = 0.933, *T*_max_ = 0.990
11236 measured reflections
3319 independent reflections
2627 reflections with *I* > 2σ(*I*)
*R*_int_ = 0.022

#### Refinement

*R*[*F* ^2^ > 2σ(*F* ^2^)] = 0.039
*wR*(*F* ^2^) = 0.105
*S* = 1.16
3319 reflections
264 parameters
H atoms treated by a mixture of independent and constrained refinement
Δρ_max_ = 0.63 e Å^−3^
Δρ_min_ = −0.83 e Å^−3^

### 

Data collection: *CrysAlis PRO* (Oxford Diffraction, 2010 ▶); cell refinement: *CrysAlis PRO*; data reduction: *CrysAlis PRO*; program(s) used to solve structure: *SIR92* (Altomare *et al.*, 1994 ▶); program(s) used to refine structure: *SHELXL97* (Sheldrick, 2008 ▶) within *WinGX* (Farrugia, 1999 ▶); molecular graphics: *PLATON* (Spek, 2009 ▶); software used to prepare material for publication: *PLATON*.

## Supplementary Material

Crystal structure: contains datablock(s) global, I. DOI: 10.1107/S1600536811021192/ng5176sup1.cif

Structure factors: contains datablock(s) I. DOI: 10.1107/S1600536811021192/ng5176Isup2.hkl

Supplementary material file. DOI: 10.1107/S1600536811021192/ng5176Isup3.cml

Additional supplementary materials:  crystallographic information; 3D view; checkCIF report

## Figures and Tables

**Table 1:** Hydrogen-bond geometry (Å, °)

*D*—H⋯*A*	*D*—H	H⋯*A*	*D*⋯*A*	*D*—H⋯*A*
N1*A*—H11*A*⋯O1*W*^i^	0.83 (2)	2.14 (2)	2.966 (2)	171 (2)
N1*A*—H12*A*⋯O12	0.89 (2)	2.07 (2)	2.914 (2)	156.8 (19)
N1*B*—H11*B*⋯O22^ii^	0.88 (2)	2.07 (2)	2.936 (2)	166.3 (19)
N1*B*—H12*B*⋯O12^iii^	0.85 (2)	2.09 (2)	2.904 (2)	162 (2)
N2*A*—H21*A*⋯O11	0.89 (3)	2.59 (3)	3.447 (2)	160 (2)
N2*A*—H21*A*⋯O12	0.89 (3)	2.35 (3)	3.125 (2)	145 (2)
N2*A*—H22*A*⋯O1*W*^iv^	0.81 (3)	2.20 (3)	3.010 (2)	175 (2)
N2*B*—H21*B*⋯O22^iii^	0.86 (3)	2.07 (3)	2.894 (2)	161 (3)
N2*B*—H22*B*⋯O11	0.91 (3)	2.09 (3)	2.880 (2)	144 (2)
N3*A*—H31*A*⋯O11^v^	0.85 (3)	2.06 (3)	2.874 (2)	159 (2)
N3*A*—H32*A*⋯O22^i^	0.84 (2)	2.19 (2)	2.923 (2)	147 (2)
N3*B*—H31*B*⋯O21	0.89 (2)	1.92 (2)	2.799 (2)	169 (2)
N3*B*—H32*B*⋯O11^vi^	0.86 (2)	2.20 (2)	2.966 (2)	149.2 (19)
O1*W*—H11*W*⋯O21	0.83 (3)	1.97 (3)	2.789 (2)	169 (3)
O1*W*—H12*W*⋯O12^vi^	0.88 (3)	1.90 (3)	2.7716 (19)	174 (3)

Symmetry codes: (i) 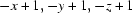; (ii) 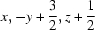; (iii) 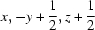; (iv) 
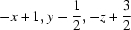
; (v) 
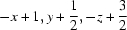
; (vi) 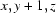.

**Fig. 1.**
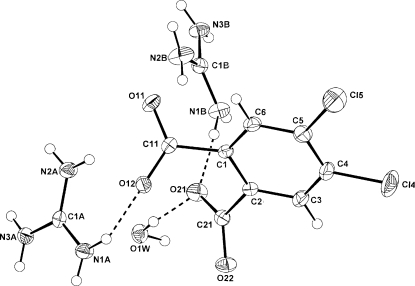
Molecular conformation and atom-numbering scheme for the two guanidinium cations, the DCPA dianion and the water molecule of solvation in (I), with inter-species hydrogen bonds shown as dashed lines. Non-H atoms are shown as 40% probability displacement ellipsoids.

**Fig. 2.**
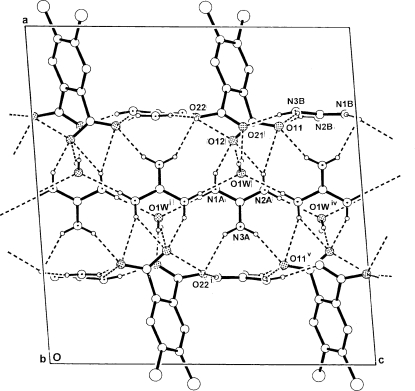
A view the two-dimensional duplex-sheet structure in the unit cell of (I), viewed down the sheets, showing hydrogen-bonding associations as dashed lines. Non-associative H atoms are omitted.

## supplementary crystallographic information

### Comment

4,5-Dichlorophthalic acid (DCPA) forms 1:1 salts with a number of Lewis bases, having most commonly low-dimensional hydrogen-bonded structures featuring the `planar' hydrogen phthalate anion (Mallinson *et al.*, 2003; Bozkurt *et al.*, 2006; Smith *et al.*, 2008, 2009; Smith & Wermuth, 2010*a*,*d*). The `nonplanar' dianionic DCPA species is much less common among the known structures, examples being the 1:2 salts with 4-ethylaniline (Büyükgüngör & Odabaşoğlu, 2007), ethylenediamine (Smith & Wermuth, 2010*c*), *n*-butylamine and piperidine (Smith & Wermuth, 2010*a*). With the strong base guanidine, the formation of 1:2 salts with dicarboxylic acids is more common, *e.g.* with phthalic acid (Krumbe & Haussuhl, 1986) and terephthalic acids (Smith & Wermuth, 2010*b*) and our 1:1 stoichiometric reaction of DCPA with guanidine carbonate not unexpectedly gave the bis(guanidinium) salt hydrate, the title compound, 2(CH_6_N_3_^+^) C_8_H_2_Cl_2_O_4_^2-^. H_2_O (I) (Fig. 1), and the structure is reported here.

In the structure of (I), the two guanidinium cations (*A* and *B*) and the water molecule of solvation provide hydrogen-bonding links between the `non-planar' DCPA dianions (Table 1). The planes of the carboxyl groups of the dianion are rotated out of the plane of the benzene ring [torsion angles C1—C2—C21–O22, -131.93 (17)°; C2—C1—C11–O11, -129.41 (16)°]. Duplex-sheet structures are formed, extending down the (011) planes in the unit cell (Fig. 2). Within these sheets there are guanidinium N—H···O_carboxyl_, N—H···O_water_ and water O—H···O_carboxyl_ associations.

### Experimental

Compound (I) was synthesized by heating together for 10 min under reflux, 1 mmol quantities of 4,5-dichlorophthalic acid and guanidine carbonate in 50 ml of 50% ethanol–water. Total evaporation of solvent gave a white non-crystalline powder which on subsequent slow room-temperature evaporation of an aqueous solution gave colourless crystalline plates of (I) from which a specimen was cleaved for the X-ray analysis.

### Refinement

H atoms potentially involved in hydrogen-bonding interactions were located by difference methods and their positional and isotropic displacement parameters were refined. Other H atoms were included at calculated positions (C—H = 0.93 Å) and treated as riding, with *U*_iso_(H) = 1.2*U*_eq_(C).

### Crystal data

**Table:**

2CH_6_N_3_^+^·C_8_H_2_Cl_2_O_4_^2^^−^·H_2_O	*F*(000) = 768
*M**_r_* = 371.19	*D*_x_ = 1.464 Mg m^−^^3^
Monoclinic, *P*2_1_/*c*	Mo *K*α radiation, λ = 0.71073 Å
Hall symbol: -P 2ybc	Cell parameters from 6093 reflections
*a* = 15.9797 (5) Å	θ = 3.2–28.6°
*b* = 6.9432 (2) Å	µ = 0.42 mm^−^^1^
*c* = 15.2266 (5) Å	*T* = 200 K
β = 94.650 (3)°	Block, colourless
*V* = 1683.84 (9) Å^3^	0.28 × 0.25 × 0.20 mm
*Z* = 4	

### Data collection

**Table:**

Oxford Diffraction Gemini-S CCD area-detector diffractometer	3319 independent reflections
Radiation source: Enhance (Mo) X-ray source	2627 reflections with *I* > 2σ(*I*)
graphite	*R*_int_ = 0.022
Detector resolution: 16.077 pixels mm^-1^	θ_max_ = 26.0°, θ_min_ = 3.2°
ω scans	*h* = −18→19
Absorption correction: multi-scan (*CrysAlis PRO*; Oxford Diffraction, 2010)	*k* = −8→8
*T*_min_ = 0.933, *T*_max_ = 0.990	*l* = −11→18
11236 measured reflections	

### Refinement

**Table:**

Refinement on *F*^2^	Primary atom site location: structure-invariant direct methods
Least-squares matrix: full	Secondary atom site location: difference Fourier map
*R*[*F*^2^ > 2σ(*F*^2^)] = 0.039	Hydrogen site location: inferred from neighbouring sites
*wR*(*F*^2^) = 0.105	H atoms treated by a mixture of independent and constrained refinement
*S* = 1.16	*w* = 1/[σ^2^(*F*_o_^2^) + (0.0562*P*)^2^ + 0.1489*P*] where *P* = (*F*_o_^2^ + 2*F*_c_^2^)/3
3319 reflections	(Δ/σ)_max_ = 0.001
264 parameters	Δρ_max_ = 0.63 e Å^−^^3^
0 restraints	Δρ_min_ = −0.83 e Å^−^^3^

### Special details

**Table:**

Geometry. Bond distances, angles *etc*. have been calculated using the rounded fractional coordinates. All su's are estimated from the variances of the (full) variance-covariance matrix. The cell e.s.d.'s are taken into account in the estimation of distances, angles and torsion angles
Refinement. Refinement of *F*^2^ against ALL reflections. The weighted *R*-factor *wR* and goodness of fit *S* are based on *F*^2^, conventional *R*-factors *R* are based on *F*, with *F* set to zero for negative *F*^2^. The threshold expression of *F*^2^ > σ(*F*^2^) is used only for calculating *R*-factors(gt) *etc*. and is not relevant to the choice of reflections for refinement. *R*-factors based on *F*^2^ are statistically about twice as large as those based on *F*, and *R*-factors based on ALL data will be even larger.

### Fractional atomic coordinates and isotropic or equivalent isotropic displacement parameters (Å^2^)

**Table:**

	*x*	*y*	*z*	*U*_iso_*/*U*_eq_	
Cl4	1.05872 (4)	0.24406 (13)	0.56212 (5)	0.0786 (3)	
Cl5	1.03264 (4)	−0.12369 (11)	0.67711 (5)	0.0676 (3)	
O11	0.70520 (8)	0.02886 (19)	0.75803 (8)	0.0304 (4)	
O12	0.66435 (8)	0.04844 (17)	0.61463 (8)	0.0260 (4)	
O21	0.70448 (9)	0.47401 (19)	0.64857 (9)	0.0325 (4)	
O22	0.73076 (9)	0.47465 (18)	0.50680 (9)	0.0313 (4)	
C1	0.80565 (11)	0.1185 (2)	0.65803 (11)	0.0207 (5)	
C2	0.81671 (11)	0.2830 (2)	0.60675 (11)	0.0221 (5)	
C3	0.89502 (12)	0.3185 (3)	0.57737 (13)	0.0338 (6)	
C4	0.96178 (12)	0.1945 (4)	0.59905 (14)	0.0397 (7)	
C5	0.95069 (13)	0.0335 (3)	0.65047 (14)	0.0361 (7)	
C6	0.87267 (12)	−0.0039 (3)	0.68007 (12)	0.0278 (6)	
C11	0.71840 (11)	0.0626 (2)	0.67974 (12)	0.0206 (5)	
C21	0.74498 (11)	0.4223 (2)	0.58557 (12)	0.0221 (5)	
N1A	0.51848 (11)	0.2730 (3)	0.54869 (12)	0.0297 (5)	
N2A	0.51734 (11)	0.2568 (3)	0.69927 (12)	0.0318 (5)	
N3A	0.40865 (10)	0.4034 (2)	0.61686 (13)	0.0281 (5)	
C1A	0.48092 (11)	0.3101 (3)	0.62137 (12)	0.0231 (5)	
N1B	0.74241 (11)	0.6136 (3)	0.96559 (11)	0.0288 (5)	
N2B	0.73486 (13)	0.3392 (3)	0.88218 (13)	0.0403 (6)	
N3B	0.74928 (11)	0.6306 (3)	0.81514 (12)	0.0314 (6)	
C1B	0.74182 (11)	0.5289 (3)	0.88781 (12)	0.0246 (6)	
O1W	0.56692 (9)	0.7224 (2)	0.63168 (9)	0.0296 (4)	
H3	0.90300	0.42630	0.54280	0.0410*	
H6	0.86520	−0.11150	0.71490	0.0330*	
H11A	0.4941 (13)	0.288 (3)	0.4989 (16)	0.029 (6)*	
H12A	0.5663 (15)	0.206 (3)	0.5529 (14)	0.040 (6)*	
H21A	0.5643 (18)	0.187 (4)	0.7004 (17)	0.056 (7)*	
H22A	0.4931 (16)	0.254 (3)	0.7442 (18)	0.048 (7)*	
H31A	0.3862 (16)	0.437 (3)	0.6634 (18)	0.049 (7)*	
H32A	0.3855 (14)	0.436 (3)	0.5679 (16)	0.038 (7)*	
H11B	0.7413 (13)	0.740 (3)	0.9689 (13)	0.033 (6)*	
H12B	0.7272 (14)	0.546 (3)	1.0077 (15)	0.037 (6)*	
H21B	0.7375 (16)	0.267 (4)	0.9278 (18)	0.055 (8)*	
H22B	0.7359 (15)	0.281 (4)	0.8289 (18)	0.056 (8)*	
H31B	0.7401 (14)	0.570 (3)	0.7640 (16)	0.039 (6)*	
H32B	0.7451 (13)	0.753 (3)	0.8189 (14)	0.032 (6)*	
H11W	0.6032 (18)	0.637 (4)	0.6354 (18)	0.058 (8)*	
H12W	0.5946 (16)	0.831 (4)	0.6270 (17)	0.054 (7)*	

### Atomic displacement parameters (Å^2^)

**Table:**

	*U*^11^	*U*^22^	*U*^33^	*U*^12^	*U*^13^	*U*^23^
Cl4	0.0240 (3)	0.1185 (7)	0.0961 (6)	0.0108 (3)	0.0218 (3)	0.0627 (5)
Cl5	0.0354 (3)	0.0869 (5)	0.0818 (5)	0.0326 (3)	0.0135 (3)	0.0423 (4)
O11	0.0375 (8)	0.0322 (7)	0.0229 (7)	−0.0065 (6)	0.0117 (6)	−0.0002 (6)
O12	0.0220 (7)	0.0265 (7)	0.0292 (7)	−0.0010 (5)	0.0003 (5)	0.0023 (5)
O21	0.0359 (8)	0.0334 (7)	0.0283 (7)	0.0100 (6)	0.0042 (6)	−0.0032 (6)
O22	0.0381 (8)	0.0279 (7)	0.0276 (7)	0.0084 (6)	0.0002 (6)	0.0072 (6)
C1	0.0228 (9)	0.0234 (9)	0.0160 (8)	0.0001 (7)	0.0024 (7)	0.0006 (7)
C2	0.0230 (9)	0.0242 (9)	0.0188 (9)	−0.0003 (7)	0.0007 (7)	0.0030 (7)
C3	0.0274 (10)	0.0399 (11)	0.0345 (11)	−0.0015 (9)	0.0050 (8)	0.0178 (9)
C4	0.0202 (10)	0.0607 (14)	0.0391 (12)	0.0016 (9)	0.0077 (9)	0.0193 (11)
C5	0.0254 (10)	0.0471 (13)	0.0360 (12)	0.0122 (9)	0.0029 (9)	0.0129 (10)
C6	0.0288 (10)	0.0299 (10)	0.0248 (10)	0.0038 (8)	0.0025 (8)	0.0082 (8)
C11	0.0241 (9)	0.0143 (8)	0.0241 (9)	0.0013 (7)	0.0059 (7)	−0.0002 (7)
C21	0.0246 (9)	0.0161 (8)	0.0251 (10)	−0.0023 (7)	−0.0004 (7)	0.0007 (7)
N1A	0.0270 (9)	0.0394 (10)	0.0230 (9)	0.0087 (8)	0.0038 (7)	0.0013 (7)
N2A	0.0275 (9)	0.0451 (10)	0.0232 (9)	0.0083 (8)	0.0051 (7)	0.0033 (8)
N3A	0.0216 (8)	0.0381 (9)	0.0248 (10)	0.0028 (7)	0.0036 (7)	−0.0012 (8)
C1A	0.0211 (9)	0.0228 (9)	0.0258 (10)	−0.0032 (7)	0.0046 (7)	0.0001 (7)
N1B	0.0426 (10)	0.0223 (9)	0.0216 (9)	−0.0005 (7)	0.0027 (7)	−0.0003 (7)
N2B	0.0696 (14)	0.0238 (9)	0.0275 (10)	−0.0036 (9)	0.0042 (9)	−0.0020 (8)
N3B	0.0434 (10)	0.0275 (10)	0.0236 (9)	0.0023 (8)	0.0048 (7)	0.0019 (7)
C1B	0.0251 (9)	0.0247 (10)	0.0237 (10)	−0.0003 (7)	0.0005 (7)	0.0011 (7)
O1W	0.0265 (7)	0.0258 (8)	0.0363 (8)	−0.0026 (6)	0.0021 (6)	0.0010 (6)

### Geometric parameters (Å, °)

**Table:**

Cl4—C4	1.725 (2)	N2B—C1B	1.324 (3)
Cl5—C5	1.728 (2)	N3B—C1B	1.326 (3)
O11—C11	1.250 (2)	N1B—H12B	0.85 (2)
O12—C11	1.265 (2)	N1B—H11B	0.88 (2)
O21—C21	1.252 (2)	N2B—H21B	0.86 (3)
O22—C21	1.256 (2)	N2B—H22B	0.91 (3)
O1W—H12W	0.88 (3)	N3B—H31B	0.89 (2)
O1W—H11W	0.83 (3)	N3B—H32B	0.86 (2)
N1A—C1A	1.326 (3)	C1—C6	1.387 (3)
N2A—C1A	1.331 (3)	C1—C11	1.510 (2)
N3A—C1A	1.321 (2)	C1—C2	1.403 (2)
N1A—H12A	0.89 (2)	C2—C21	1.514 (2)
N1A—H11A	0.83 (2)	C2—C3	1.385 (3)
N2A—H22A	0.81 (3)	C3—C4	1.390 (3)
N2A—H21A	0.89 (3)	C4—C5	1.384 (3)
N3A—H32A	0.84 (2)	C5—C6	1.384 (3)
N3A—H31A	0.85 (3)	C3—H3	0.9300
N1B—C1B	1.322 (3)	C6—H6	0.9300
			
H11W—O1W—H12W	105 (3)	C2—C3—C4	120.62 (19)
H11A—N1A—H12A	118 (2)	C3—C4—C5	120.24 (19)
C1A—N1A—H12A	119.0 (14)	Cl4—C4—C3	119.43 (19)
C1A—N1A—H11A	121.8 (15)	Cl4—C4—C5	120.33 (17)
H21A—N2A—H22A	115 (2)	Cl5—C5—C4	120.90 (16)
C1A—N2A—H22A	123.6 (18)	Cl5—C5—C6	119.38 (16)
C1A—N2A—H21A	118.3 (17)	C4—C5—C6	119.71 (19)
C1A—N3A—H32A	120.1 (16)	C1—C6—C5	120.30 (18)
H31A—N3A—H32A	119 (2)	O12—C11—C1	115.63 (15)
C1A—N3A—H31A	121.1 (17)	O11—C11—O12	125.16 (16)
C1B—N1B—H12B	116.8 (15)	O11—C11—C1	119.18 (16)
H11B—N1B—H12B	120 (2)	O22—C21—C2	117.60 (15)
C1B—N1B—H11B	119.8 (13)	O21—C21—C2	116.69 (15)
C1B—N2B—H22B	119.6 (18)	O21—C21—O22	125.71 (16)
C1B—N2B—H21B	122.2 (19)	C2—C3—H3	120.00
H21B—N2B—H22B	118 (3)	C4—C3—H3	120.00
H31B—N3B—H32B	122 (2)	C5—C6—H6	120.00
C1B—N3B—H31B	117.4 (14)	C1—C6—H6	120.00
C1B—N3B—H32B	117.4 (14)	N1A—C1A—N2A	119.67 (18)
C6—C1—C11	119.89 (14)	N1A—C1A—N3A	120.31 (18)
C2—C1—C6	120.26 (16)	N2A—C1A—N3A	120.00 (18)
C2—C1—C11	119.44 (15)	N1B—C1B—N2B	119.68 (19)
C3—C2—C21	120.38 (15)	N1B—C1B—N3B	121.1 (2)
C1—C2—C21	120.74 (15)	N2B—C1B—N3B	119.24 (19)
C1—C2—C3	118.86 (16)		
			
C6—C1—C2—C3	1.2 (2)	C1—C2—C21—O21	47.5 (2)
C6—C1—C2—C21	−177.34 (16)	C1—C2—C21—O22	−131.93 (17)
C11—C1—C2—C3	−171.43 (16)	C3—C2—C21—O21	−131.02 (18)
C11—C1—C2—C21	10.0 (2)	C3—C2—C21—O22	49.6 (2)
C2—C1—C6—C5	−1.1 (3)	C2—C3—C4—Cl4	−179.47 (15)
C11—C1—C6—C5	171.54 (17)	C2—C3—C4—C5	0.0 (3)
C2—C1—C11—O11	−129.41 (16)	Cl4—C4—C5—Cl5	−1.3 (3)
C2—C1—C11—O12	52.6 (2)	Cl4—C4—C5—C6	179.63 (16)
C6—C1—C11—O11	57.9 (2)	C3—C4—C5—Cl5	179.30 (17)
C6—C1—C11—O12	−120.04 (17)	C3—C4—C5—C6	0.2 (3)
C1—C2—C3—C4	−0.7 (3)	Cl5—C5—C6—C1	−178.78 (15)
C21—C2—C3—C4	177.87 (18)	C4—C5—C6—C1	0.4 (3)

### Hydrogen-bond geometry (Å, °)

**Table:**

*D*—H···*A*	*D*—H	H···*A*	*D*···*A*	*D*—H···*A*
N1A—H11A···O1W^i^	0.83 (2)	2.14 (2)	2.966 (2)	171 (2)
N1A—H12A···O12	0.89 (2)	2.07 (2)	2.914 (2)	156.8 (19)
N1B—H11B···O22^ii^	0.88 (2)	2.07 (2)	2.936 (2)	166.3 (19)
N1B—H12B···O12^iii^	0.85 (2)	2.09 (2)	2.904 (2)	162 (2)
N2A—H21A···O11	0.89 (3)	2.59 (3)	3.447 (2)	160 (2)
N2A—H21A···O12	0.89 (3)	2.35 (3)	3.125 (2)	145 (2)
N2A—H22A···O1W^iv^	0.81 (3)	2.20 (3)	3.010 (2)	175 (2)
N2B—H21B···O22^iii^	0.86 (3)	2.07 (3)	2.894 (2)	161 (3)
N2B—H22B···O11	0.91 (3)	2.09 (3)	2.880 (2)	144 (2)
N3A—H31A···O11^v^	0.85 (3)	2.06 (3)	2.874 (2)	159 (2)
N3A—H32A···O22^i^	0.84 (2)	2.19 (2)	2.923 (2)	147 (2)
N3B—H31B···O21	0.89 (2)	1.92 (2)	2.799 (2)	169 (2)
N3B—H32B···O11^vi^	0.86 (2)	2.20 (2)	2.966 (2)	149.2 (19)
O1W—H11W···O21	0.83 (3)	1.97 (3)	2.789 (2)	169 (3)
O1W—H12W···O12^vi^	0.88 (3)	1.90 (3)	2.7716 (19)	174 (3)

Symmetry codes: (i) −*x*+1, −*y*+1, −*z*+1; (ii) *x*, −*y*+3/2, *z*+1/2; (iii) *x*, −*y*+1/2, *z*+1/2; (iv) −*x*+1, *y*−1/2, −*z*+3/2; (v) −*x*+1, *y*+1/2, −*z*+3/2; (vi) *x*, *y*+1, *z*.
